# Pragmatics: Exploring language use by younger generations in Pedi families

**DOI:** 10.4102/sajcd.v72i1.1097

**Published:** 2025-08-26

**Authors:** Mamaila J. Mogolane, Joanne Neille, Jenna Sher

**Affiliations:** 1 Department of Speech Pathology and Audiology, Faculty of Humanities, University of the Witwatersrand, Johannesburg, South Africa; 2 Department of Rehabilitative and Natural Sciences, Faculty of Health Sciences, University of Fort Hare, East London, South Africa

**Keywords:** Pedi, culture, pragmatics, verbal language, non-verbal language, speech-language pathologists, families, cultural norms

## Abstract

**Background:**

The people of the Pedi culture place great value on, and take pride in, adhering to their culture, as reflected in the manner in which they communicate verbally and non-verbally. However, little is documented about the ways in which verbal and non-verbal language is used socially by the younger generations in the Pedi culture.

**Objectives:**

This article examines how verbal and non-verbal social language skills and functions are used by the younger generations in Pedi families.

**Method:**

A qualitative research design was employed, underpinned by the principles of direct participant observations and semi-structured interviews. A total of 22 participants from seven families were included, and the data were analysed through thematic analysis.

**Results:**

The results showed how younger generations in Pedi families respectfully execute verbal and non-verbal social language skills and functions.

**Conclusion:**

Understanding the cultural nuances of communication in the Pedi culture, including respect expressed through verbal and non-verbal cues, is critical for speech-language pathologists (SLPs). By recognising these cultural practices as differences rather than deficits, SLPs can provide more effective, respectful and culturally appropriate interventions.

**Contribution:**

The findings of this study contribute to a broader understanding of how language is used in the Pedi culture and offer valuable insights for practitioners working with clients from diverse cultural backgrounds. Moreover, these findings call for a more inclusive approach to pragmatics in speech-language pathology that acknowledges the rich diversity of communication practices worldwide.

## Introduction and literature review

About 5.9 million South Africans are believed to be native Sepedi speakers (Census 2022b). Sepedi ranks as the fourth most commonly spoken native language in South Africa (Census, 2022b). Pedi people typically reside in the provinces of Limpopo, Mpumalanga and Gauteng, but the majority of them are inhabitants of the Limpopo province (Census, 2022b). Their family ties are strengthened as a result of their traditional values being passed down from generation to generation (Lebaka, 2019; Makgabo, 2021). Younger generations of the Pedi culture are taught cultural norms; these include, knowing how and when to speak to their different family members (Makgabo, 2021; Mphasha et al., 2021). Little is however documented about the ways in which verbal and non-verbal language is used socially by younger generations in the Pedi culture. Younger generations and younger people in this study were participants identified as daughters, sons, a nephew, grandchildren and siblings (refer to Table 1). The terms were used to describe their familial positions, not age, within their family structure.

**TABLE 1:** Participants demographics.

Family identity	Number of family members	Position of family member	Pseudonym	Gender	Age (years)	Education or employment status	Main locale of observations	Locale of interviews
Family one	Three	Grandfather		Male	66	Pensioner	Outside	
		Grandmother	Mogošadi	Female	63	Pensioner		Inside
		Grandson	Mokone	Male	19	Matriculated (Gap year)		Inside
Family two	Four	Mother	Mologadi	Female	69	Pensioner	Outside	Inside
		Daughter/Sister	Hunadi	Female	43	Unemployed		Inside
		Son/Brother		Male	35	Unemployed		
		Son/Brother		Male	28	Unemployed		
Family three	Three	Grandmother		Female	77	Pensioner	Outside	
		Grandmother’s caregiver	Noko	Female	41	Employed by family		Inside
		Grandson		Male	5	Kindergarten		
Family four	Three	Grandfather/Husband	Mphele	Male	50	Employed	Inside	Inside
		Grandmother/Wife	Mosebo	Female	48	Employed		Inside
		Granddaughter		Female	14	High school (Grade 8)		
Family five	Three	Grandmother/Mother		Female	62	Pensioner	Inside	
		Mother/Daughter	Mosebjadi	Female	37	Employed		Inside
		Granddaughter/Daughter		Female	6	Preschool		
Family six	Three	Grandmother	Monene	Female	52	Employed	Inside	Inside
		Grandson		Male	13	Primary school (Grade 7)		
		Granddaughter		Female	15	High school (Grade 9)		
Family seven	Three	Older sister/Aunt		Female	43	Employed	Outside	
		Younger sister/Mother	Mokgadi	Female	38	Unemployed		Inside
		Son of the younger sister/Nephew		Male	10	Primary school (Grade 4)		

Source: Adapted from Mogolane, M.J. (2024). *An exploration into the verbal and non-verbal social use of the Sepedi language in the Pedi culture*. Master’s dissertation. University of the Witwatersrand. Retrieved from https://hdl.handle.net/10539/44803

Pragmatics, the study of language use in various social contexts, emphasises cultural influences (Feruza, 2024; Khodeir et al., 2017; Kohnert et al., 2020). Context is essential in deducing meaning from verbal or non-verbal communication (Ekoro & Gunn, 2021). Pragmatics and context are thus inextricably linked (Ekoro & Gunn, 2021). It is important to note that context does not only allude to physical settings like homes but also to the linguistic context which describes the unique ways that languages are used in different cultures, covering domains such as differences in grammatical structure and differences in the performance of speech acts (Ekoro & Gunn, 2021). Verbal communication involves speech acts, narrative skills and social reciprocity, while non-verbal communication includes body language, facial expressions and eye contact (Alduais et al., 2022; American Speech Language Hearing Association [ASHA], n.d.; Tomasello, 2023). South African speech-language pathologists (SLPs) must evaluate the presence of pragmatic language impairments. However, if the cultural rules of a language are not taken into account, misdiagnosis may occur, resulting in inappropriate interventions and poor adherence to home programmes. Traditionally, SLPs working in non-Western contexts have relied on the use of Western assessment and intervention tools because of a lack of contextually relevant, standardised tools within our own contexts. This emphasises the need for culturally, contextually and linguistically relevant materials to ensure accurate evaluation and effective intervention (Vorster et al., 2022).

African language use conventions, both verbal and non-verbal, differ from culture to culture as well as from Western conventions in certain ways and are comparable in others (Moloko-Phiri et al., 2022; Nenungwi, 2015; Wojtowicz, 2021). African clients may use language in ways that differ from the expectations of pragmatics evaluation instruments and from the ethnicity of SLPs providing them with services. For example, their interactions with older individuals may follow culturally specific conventions (Moloko-Phiri et al., 2022). Nenungwi (2015) stated that, in the Venḓa culture, it is considered disrespectful for younger people to look an adult in the eyes when they are conversing with them. According to Mncwango (2009) and Vorster et al. (2022), it is also required of Pedi children to avoid eye contact as a show of respect for their elders. Older male Xhosa and Zulu clients may anticipate being referred to as *tata* [father] or *baba* [father] rather than by their names as a form of reverence (Moloko-Phiri et al., 2022; Ntuli, 2012). In the Sotho and Tswana cultures, elders are also addressed using titles such as *rra* [Mr/father] or *mme* [Mrs/mother] rather than their names (Mudau et al., 2024; Ngoasheng, 2006). It is considered impolite and disrespectful to not use these titles (Mudau et al., 2024). In the Tswana and Venḓa cultures, when greeting someone older than themselves, children clap or put their hands together in a respectful and humble manner (Bagwasi, 2012; Nenungwi, 2015). Social hierarchy is also embedded in grammar where certain prefixes or plural forms may be used by younger generations when communicating with their elders to indicate respect, rather than a literal number, such as in the Tswana and Venḓa cultures (Bagwasi, 2012; Hellen, 2018).

Certain actions, such as indirect eye contact as a sign of respect, may be perceived as socially inappropriate in other contexts and cultures (ASHA, n.d.). Contrary to African contexts, in Western contexts, such as in the United States of America, France and in the German culture, maintaining direct eye contact during a conversation is desired as it is seen as a sign of trustworthiness, genuine engagement and sincerity (Aleksandrovna, 2021; Bonaccio et al., 2016; Elhadji, 2017). According to Al-Qaderi et al. (2017) and Bonaccio et al. (2016), people in Western cultures view indirect eye contact as being unfriendly, disrespectful and sometimes as a sign of pragmatic difficulty. The majority of Americans do not interpret pointing at someone negatively (Aleksandrovna, 2021). In contrast, native Americans consider this pointing gesture to be very disrespectful (Aleksandrovna, 2021). In many African parts of the world, it is also frowned upon (Al-Qaderi et al., 2017). Given these and other disparities (e.g. performance of greetings), a lack of understanding pertaining to how language is used by clients from different African cultures runs the risk of endangering both the effectiveness of speech-language pathology services and the morality of requiring families to use methods that are not in line with their own culture (Aleksandrovna, 2021; Health Professions Council of South Africa [HPCSA], 2019; Pascoe & Norman, 2011).

In order to provide culturally congruent pragmatics evaluations and interventions for African clients, SLPs must be aware of, and understand their different cultural executions of verbal and non-verbal language use, and not flag them as a disorder as per the criteria of a Westernised tool, when in actual fact, it is a difference (HPCSA, 2019; Moloko-Phiri et al., 2022; Salins et al., 2023). A pragmatics evaluation must be able to account for cultural differences (e.g. accepting with both hands to demonstrate respect) and linguistic differences (e.g. communicating in the plural form) (Jodache et al., 2019; Salins et al., 2023). It should be practical, attentive to the vast range of social norms that are accepted within and across communities and cultures, and entail the cooperative efforts of families (ASHA, n.d.; HPCSA, 2019; Salins et al., 2023). Given that intervention is an ongoing process that extends into the homes of clients by way of home programme activities, it must also support a culturally safe, family-centred approach that aligns with their cultural beliefs and values (Bornman & Louw, 2021; Khoza-Shangase & Mophosho, 2018; Salins et al., 2023). A non-functional intervention that lacks relevance to the client, and hinders their ability to participate normally in their daily life activities, is deemed ineffective (Kohnert et al., 2020).

The importance of pragmatics in speech-language pathology necessitates an understanding of culturally specific pragmatic norms (Cummings, 2021). However, there remains a paucity of research on pragmatic language use in African cultures, particularly within the Pedi culture. As noted by Ameka and Terkourafi (2019) and Harricharan et al. (2011), pragmatic language skills are often implicitly treated as culturally neutral, with dominant theories and models primarily based on communicative behaviours observed in Western contexts. This presents several challenges for SLPs when applying Western-based communication models to clients from non-Western cultural backgrounds (Harricharan et al., 2011). Pragmatic competence should therefore reflect the variation in language use patterns between Western and non-Western contexts (Ameka & Terkourafi, 2019). In many African cultures, such as among the Pedi, communication is deeply embedded in cultural values and social hierarchies, with both verbal and non-verbal behaviours carrying specific meanings. For example, forms of greeting, turn-taking, eye contact, the use of silence and gestures such as hand-clapping or avoiding direct eye contact with elders are culturally informed and context-dependent. The field of speech-language pathology places a high value on cultural responsiveness, which includes recognising and respecting these diverse communicative behaviours in assessment and intervention practices (Bakić-Mirić et al., 2018; Moloko-Phiri et al., 2022). In light of this, the objective of this study was to explore how verbal and non-verbal social language skills and functions are used in the home context by younger generations in Pedi families.

## Research methods and design

### Research context

The research was conducted at Mahwelereng Zone 1, in the Mogalakwena Municipality, located in the Limpopo province of South Africa. Mahwelereng is a township that was founded in 1964 on the Makalakalakop ranch, with a total population of 41 072 people and 10 874 households (Census, 2022a). Over 99% of the population is made up by the Black population, while 0.6% is shared by the Coloured, Indian or Asian, and White ethnicities (Census, 2022a). Native Sepedi speakers constitute 75.7% of the population (Census, 2022a), thus making the Pedi culture the culture of dominance in Mahwelereng. This research was conducted from an emic perspective (Verbuyst, 2024), which involved the first author being from the Pedi community.

### Research design

A qualitative research design was employed, underpinned by the principles of direct participant observations and semi-structured interviews. Observations took an inductive approach while semi-structured interviews took a deductive approach (Azungah, 2018). The observations and interviews were conducted using a self-developed observation guideline and an interview schedule that were created based on the work of Khodeir et al. (2017). The pragmatic language skills and pragmatic language functions subtests of the Egyptian Arabic Pragmatic Language Test (Khodeir et al., 2017) were utilised in the development of the observation guideline and interview schedule, aligning with the research objective. Furthermore, as in the Arabic culture, the language skills and functions reflected in these subtests are relevant to the Pedi context, as they are similarly embedded in the social use of the Sepedi language within the Pedi culture.

### Sampling methods and participants

Non-probability purposive sampling was utilised in this study. Pedi families from Mahwelereng Zone 1 were intentionally selected as the sample for this research (Crossman, 2017; Elfil & Negida, 2017). The families were approached for potential participation through an information session gathering that was arranged with the assistance of the ward councillor, where the study’s objective was explained. Participants were not given any examples, either during the information session or in the participant information sheets, that could have influenced or primed their use of verbal and non-verbal communication in expected ways. A total number of 22 participants, from seven families, comprising of a minimum of three family members, spanning different genders and generations were the sample size. None of the family members from the families that participated indicated that they had been diagnosed with communication impairments when asked. Families with family members diagnosed with any communication impairments were going to be excluded from this research study to avoid drawing incorrect conclusions about the social use of the Sepedi language in the Pedi culture (i.e. concluding that it is a Pedi cultural norm, when in fact, the family member is being communicated to in that way because of their condition).

Table 1 provides a description of the participants. The description includes the number of family members, the positions that they hold within the family, their genders, ages, employment or education status, the main location where observations were conducted in their home, as well as the location where the individual semi-structured interviews were conducted in their home (i.e. inside or outside their house). Indicating the location of data collection contributes to the rigour and replicability of the study. It allows readers to better interpret findings and understand how the setting may have influenced participants’ responses or behaviours. There is a specification for family members who hold multiple positions in the family, in relation to their family members. Moreover, the location of the interviews and pseudonyms are only written next to the family member who participated in the semi-structured interviews. The pseudonyms drew inspiration from praise names (Maahlamela, 2017) which people of the Pedi culture utilise when they respectfully refer to each other. All family members from the seven families participated in the observations, hence, the location is not specified per participant as with the interview locations.

### Direct participant observations

The families were observed in their homes while interacting, with a special focus on how the younger generations used verbal and non-verbal language with their elders. To maintain the naturalistic factor and to avoid causing discomfort during observations, the researcher engaged with the participants as a casual visitor (i.e. an active overt direct participant as an observer). In essence, the researcher’s main identity in the setting was as a participant, but they were also collecting data openly. These observations lasted for 4 h each day, over the course of 3 days, for each family. There were two observation sessions per day, a morning session (09:00–13:00), and an afternoon session (14:00–18:00), with two different families. The morning session was for families that did not have work or school commitments (i.e. families with family members who have retired, are unemployed or are taking a gap year). The afternoon session was for families that had work or school commitments and were only available in the afternoon. This structure of the observations, as well as the times, was suggested by the community members who attended the information session. With the observation guideline on hand, field notes were taken. All participants (*n* = 22) partook in the observations.

### Semi-structured interviews

Willing family members (18 years and older) were interviewed on how non-verbal and verbal social skills and functions are used in the Pedi culture, specifically in the home context by younger generations. The individual semi-structured interviews were conducted with 10 (*n* = 10) of the participants, inside one of the rooms in their homes, for confidentiality purposes. After the 3-day cycle of observations was completed with the two different families, the fourth day was allocated for the semi-structured interviews, with participants from the two families. After completing the semi-structured interviews, the cycle started again with the next two families until all seven families were observed and interviewed. Similar to the observations, the interviews also had a morning session and an afternoon session. The allocated time for the interviews was 25 min to an hour. The interviews were conducted in Sepedi and transcribed in Sepedi. Data were analysed in Sepedi and then translated into English. Transcribing and analysing the data in Sepedi helped in maintaining the authenticity of the data, and it honoured the participants’ voices, ensuring that their perspectives were not filtered through the English language. The content was not translated word for word, however, the translations conveyed the meaning that was intended by the participants (Harmon, 2019). It was ensured that translations were accurate through member checking. The translations were reviewed by the participants to ensure preservation of their voice, and to correct any misunderstandings. After analysis, interpretations and conclusions, a draft report of the gathered data was shared with the participants and their feedback was solicited regarding the accuracy and veracity of the data (Rose & Johnson, 2020).

### Data analysis

Concurrent with data collection, a thematic analysis of the data collected during observations and interviews was conducted, utilising both inductive and deductive measures (Braun & Clarke, 2021; Clarke & Braun, 2017; Proudfoot, 2023). Table 2 depicts the main themes and sub-themes that were derived from thematic analysis.

**TABLE 2:** Main themes and sub-themes derived from thematic analysis.

Main themes	Sub-themes
1. Respect as a core component of communication	1.1 Gestures and body language in communication 1.2 Use of plural forms in communication 1.3 Greetings in the Pedi culture 1.4 Direct and indirect eye contact 1.5 Turn-taking and silence
2. Verbal language use	2.1 Requesting and informing 2.2 Initiating and providing input in conversations

### Ethical considerations

Ethical approval to conduct the study was obtained from the University of the Witwatersrand Research and Ethics Committee Non-Medical on 18 August 2023. The ethical clearance number is H23/08/28. Permission to conduct the research was also sought from the ward councillor, who serves as the official representative of the community within the local government. The approval of the ward councillor was essential in ensuring that the research aligns with community interests, respects local protocols and does not disrupt existing initiatives or daily life in the township. Moreover, involving the ward councillor assisted in fostering trust and cooperation from the residents, thereby enhancing the credibility and ethical integrity of the research process. Before the observations and interviews, informed verbal assent, informed written assent and informed written consent were secured from all participants as per the university’s ethical guidelines for applicants. Also, all age groups of participants had their own version of the participant information sheet that explained the aim of the study, as stipulated in the guidelines. All documents had English and Sepedi versions. To ensure the anonymity and confidentiality of participants, pseudonyms were used in place of real names throughout the processes of data collection, analysis and reporting. Interviews were conducted in private spaces within participants’ homes to promote comfort and privacy. Furthermore, to minimise the risk of unauthorised access to sensitive data, audio recordings were transcribed in a secure, private environment using earphones. These measures were taken to uphold ethical standards and safeguard participants’ privacy and dignity.

## Results

The results highlight the key themes and sub-themes derived from the study, focusing on how respect is embedded in communication within Pedi families. Respect as a core component of communication is demonstrated through various non-verbal and verbal cues, including gestures (e.g. clapping and removing headgear), eye contact (indirect eye contact as a sign of respect) and structured turn-taking (younger generations listening more than speaking). The use of plural forms when addressing elders further illustrates these respectful interactions. Verbal language use focuses on pragmatic language skills such as requesting and informing where younger individuals follow structured communication channels, often relaying information through their mothers before it reaches the head of the household, reinforcing traditional hierarchies. Overall, the findings emphasise that respect by younger generations is a fundamental principle shaping communication in the Pedi culture.

### Respect as a core component of communication

In the Pedi culture, respect is communicated through a combination of gestures, body language and verbal language that demonstrate deference to elders. The behaviours and gestures that emerged in this study highlight how deeply respect is woven into daily interactions, particularly between younger and older generations. They are an integral part of communication, playing a crucial role in social cohesion and the transmission of cultural values across generations.

#### Gestures and body language in communication

In the Pedi culture, gestures and body language are central to expressing respect, especially in interactions between younger generations and their elders. Common gestures include clapping, holding and kissing hands, as well as bending the knee, removing headwear and refraining from pointing fingers. These actions are used in various combinations, depending on the context. For example, younger people greet their elders by clapping their hands and elders often respond by holding or kissing their hands.

It was observed that all of the younger generations across the seven families would clap their hands when greeting family members who were older than them. Their elders would then hold and kiss their hands to accept their greeting. This gesture signifies respect and acknowledges the status of the elder:

‘… Clapping your hands is a sign of respect to the older person that you are greeting.’ (Monene, grandmother, family six)

Kneeling while serving food or accepting gifts from elders is another significant sign of respect. In the families observed, younger generations would kneel when giving food to or receiving items from elders, signalling humility and respect:

‘When you bring their food, you will bring them water to wash their hands first and you kneel as you give them, then give them a towel to dry their hands. You will then come back with their food, kneel, then hand them their food.’ (Mosebjadi, mother/daughter, family five)

Accepting gifts or items from elders with two open hands was another observed gesture that conveyed respect. This behaviour was observed across multiple families, and participants indicated that accepting with both hands, clapping and verbally relaying gratitude is considered proper etiquette in the Pedi culture:

‘We accept with both our hands when being given something.’ (Monene, grandmother, family six)

‘For example, if my mother gives you R2 to buy an orange, you must not grab it from her, you must clap your hands and say “thank you, grandmother” to show respect.’ (Hunadi, daughter/sister, family two)

Taking off of hats and caps when in the home environment and when greeting was observed, and it was performed by the younger males in the families. It is customary for male members of the Pedi community to remove their hats when entering the home or greeting others. This gesture signifies deference and respect for the people they interact with:

‘In the Pedi culture, when men greet, they take off their headgear. That is how a Pedi man is recognised.’ (Monene, grandmother, family six)

‘When a Pedi man interacts with people, he takes off his headgear. That shows respect.’ (Hunadi, daughter/sister, family two)

‘Inside the house men totally do not wear headgear.’ (Mokgadi, younger sister/mother, family seven)

Pointing fingers at elders is seen as highly disrespectful in the Pedi culture. Younger people are taught to avoid such gestures during interactions with those older than them:

‘When speaking to a person that is older than you, you must not point fingers at them.’ (Noko, caregiver, family three)

#### Use of plural forms in communication

The use of plural forms reflects respect in the Pedi culture. When addressing elders, younger people are expected to use plural forms, even when referring to a single elder. This practice shows the humility and respect that younger generations must demonstrate towards their elders. In interactions, younger people would respond to older family members in the plural form by saying ‘šee’, instead of ‘hee’ which is a singular form of response. The use of the plural prefix *bo-* [many] before titles (e.g. *bomma* for mother, *borakgolo* for grandfather), is also a sign of respect:

‘Pedi people are close, we call each other *bosesi* [sisters] and *bobuti* [brothers]. We humble ourselves, we address each other in the plural form by saying *bommane* [aunts], or *bosesi* [sisters].’ (Mosebo, grandmother/wife, family four)

Younger generations were observed to consistently address their elders in the plural form, even if only one person was being referred to. This was confirmed by one participant, who said, ‘You cannot address a person that is older than you in the singular form, you must address them in the plural form as if you are generalising’ (Noko, caregiver, family three).

Table 3 outlines how the prefix *bo-* [many] was utilised by the younger generations, and to whom it was directed to in the family.

**TABLE 3:** Insertion of the Sepedi prefix *bo-* [many] when younger generations addressed their elders.

Family identity	Title of family members relative to the younger generations	Addresser and addressee
Family one	*Rakgolo* [Grandfather] *Koko* [Grandmother]	***Bo****rakgolo*- Grandson to his grandfather ***Bo****koko*- Grandson to his grandmother
Family two	*Mma* [Mother]	***Bo****mma*- All children to their mother
Family three	*Koko* [Grandmother]	***Bo****koko*- Grandson to both his grandmother and his grandmother’s caregiver
Family four	*Rakgolo* [Grandfather] *Koko* [Grandmother]	***Bo****rakgolo*- Granddaughter to her grandfather ***Bo****koko*- Granddaughter to her grandmother
Family five	*Koko* [Grandmother] *Mma* [Mother]	***Bo****koko*- Granddaughter to her grandmother ***Bo****mma*- Daughter to her mother
Family six	*Koko* [Grandmother]	***Bo****koko*- Grandson and granddaughter to their grandmother
Family seven	*Mma* [Mother] *Mmamogolo* [Aunt]	***Bo****mma*- Son to his mother ***Bo****mmamogolo*- Nephew to his aunt

Source: Adapted from Mogolane, M.J. (2024). *An exploration into the verbal and non-verbal social use of the Sepedi language in the Pedi culture*. Master’s dissertation. University of the Witwatersrand. Retrieved from https://hdl.handle.net/10539/44803

#### Greetings in the Pedi culture

Greetings in the Pedi culture are not simply a formality but are deeply ingrained with respect for elders. Greetings are often performed respectfully using a combination of non-verbal gestures and verbal phrases. Younger generations greeted their elders by clapping hands, kneeling and using the plural form in their speech. These gestures were performed in conjunction with the verbal greeting formula: ‘re a lotšha’ [we are greeting] followed by ‘agee, le kae?’ [I accept the greeting, how are you?], to which the younger generations responded with ‘re gona’ [we are fine]. To validate these findings, participants shared on the greeting formula that the younger generations use when respectfully greeting people who are older than them:

‘Younger people should greet older people by clapping their hands, bending their knee, humbling themselves, and by saying “thobela” which is a formal way of greeting. It is up to you if you want to utilise thobela or say “we are greeting”.’ (Mologadi, mother, family two)

The elders typically ask how the younger ones are, not conversely, signalling their acceptance of the greeting:

‘If I greet an older person, it is up to them to decide how they reply, but in most cases they are the ones that ask us how we are.’ (Mokone, grandson, family one)

It is customary for younger people to initiate greetings when encountering their elders. Older people do not greet younger people first; this is seen as a breach of cultural etiquette. One participant remarked:

‘In the Pedi culture, an older person must not greet a younger person first, it must be vice versa.’ (Mokone, grandson, family one)

In contrast, some participants relayed that the initiation of greetings is not solely the responsibility of the younger generations. Older people can also initiate greetings when they come across younger people:

‘No, even us older people greet the younger generations, for example, “hello Joy”. It is not a burden that must be carried by the younger generations only.’ (Hunadi, daughter/sister, family two)

#### Direct and indirect eye contact

The Pedi culture places great emphasis on the role of eye contact in communication. While direct eye contact is a sign of intimacy and closeness among peers as observed between the siblings from family two when communicating, for instance, it is a different matter when it comes to interactions between younger and older generations. Indirect eye contact was observed as a sign of respect when conversing with elders. Direct eye contact, especially with older males, is seen as defiant and disrespectful:

‘According to our culture, you cannot maintain eye contact with someone that is superior to you.’ (Mokone, grandson, family one)

‘Older people do not feel comfortable if they speak to a person younger than them and the younger person stares. The younger person must show respect by not staring and not maintaining eye contact. They were raised to not look at a person, especially a man, directly in the eyes because it comes across as disrespect.’ (Mokgadi, younger sister/mother, family seven)

#### Turn-taking and silence

Turn-taking during conversations is another area where respect is manifested in the Pedi culture. Younger people are expected to listen attentively and refrain from interrupting or speaking out of turn, especially when elders are speaking. When elders reprimand younger generations, the latter are expected to remain silent and listen. Interrupting or attempting to defend oneself is seen as disrespectful:

‘You are not allowed to have a dialogue with a person that is older than you, they do not want that as it comes across as disrespect, you must listen. You can speak when they give you the chance to speak. When they are speaking, you must be quiet.’ (Mokgadi, younger sister/mother, family seven)

Silence is expected from younger generations during family mealtimes and family TV time:

‘We do not converse when we eat, it will be silent. The elders may sometimes talk amongst themselves, but the younger ones do not talk.’ (Mokgadi, younger sister/mother, family seven)

‘We do not speak while watching TV.’ (Noko, caregiver, family three)

### Verbal language use

Verbal language use within Pedi families reflects deeply rooted cultural norms that guide communication roles and patterns across generations. These norms emphasise respect, hierarchy and structured interaction, particularly in family contexts.

#### Requesting and informing

Communication between parents and children often follows a specific order. Children typically inform their mothers of something, who then relay the information to the father. This structure of communication ensures that authority is respected and maintained within the family. For example, the granddaughter from family four relayed a message from school to her grandmother, then her grandmother passed the message to the grandfather. This was the same for the grandson from family one when he relayed a message from a neighbour. It was therefore noted that children in the Pedi culture have channels of communication that they have to follow when informing or requesting for something:

‘Children must request for things such as new shoes from the mother, then the mother will relay the request to the father.’ (Mosebo, grandmother/wife, family four)

‘All children will inform the mother, and the mother will tell the father, that is the procedure that I know.’ (Mosebjadi, mother/daughter, family five)

‘Children must tell the mother, and the mother will tell the father. If a child goes straight to the father, it will be disregarding their culture, and it will give the mother the impression that the child loves the father more than her.’ (Monene, grandmother, family six)

Noko (caregiver, family three) shared that informing a parent about something or requesting something from a parent by a child is gender-specific. Female children can speak with their mother, while male children can speak with their father:

‘That one is gender-specific. A male child will go to the father, while a female child will go to the mother. A female child cannot go to her father and tell him information that is female related.’

#### Initiating and providing input in family conversations

In terms of initiating and commenting on conversations, it was observed that younger generations in Pedi families are expected to follow a more passive role. The initiation of topics is typically introduced by elders, while younger members are less likely to participate unless prompted. This is particularly true when elders are engaged in conversations among themselves, as younger people are expected to remain silent and not chime in when their elders are having a conversation:

‘Children should not converse with their elders. They must let them be, and go play with their friends.’ (Mologadi, mother, family two)

‘In the Pedi culture, a child is not allowed to comment in on elders’ conversations. A child must be quiet, and not comment because that will be a problem.’ (Mokgadi, younger sister/mother, family seven)

In the Pedi culture, respect is not merely a passive value but an active, observable practice that permeates both verbal and non-verbal communication. From the use of gestures like hand-clapping and kneeling to the application of plural forms of address, every aspect of interaction with elders is governed by a deep sense of respect. The rules regarding eye contact, turn-taking and silence further highlight the importance of deference to authority, especially when interacting with older family members. These cultural practices help reinforce social hierarchies, ensuring that younger generations understand their roles and responsibilities within the family structure. Through these various forms of communication, the Pedi culture teaches younger generations to respect their elders and to maintain a harmonious, well-ordered social structure. Whether through specific greeting formulas, or the avoidance of direct eye contact, respect remains a central pillar of social interaction in Pedi communities.

## Discussion

To provide culturally sensitive services to African clients, SLPs must go beyond simply acquiring vocabulary and develop an understanding of the cultural norms that inform respectful verbal and non-verbal language use, particularly among younger generations in the Pedi culture, where communication reflects deeply rooted traditions of respect, such as clapping hands when greeting elders or avoiding direct eye contact as a sign of deference (HPCSA, 2019; Moloko-Phiri et al., 2022). Significant factors that surfaced in this study are discussed in relation to the implications for speech-language pathology assessment and intervention.

### Gestures and body language as respect in the Pedi culture

In the Pedi culture, non-verbal cues such as bowing, kneeling and bending the knee are commonly used to show respect (Mphasha et al., 2021). Such gestures, while shared across various African cultures, have distinct interpretations based on context. For instance, while kneeling is common in West Africa (Wojtowicz, 2021), it also holds significant meaning within the Pedi culture as a gesture of respect, especially when interacting with elders. Similarly, other cultures such as the Tswana, Venḓa and Hindu cultures also emphasise the importance of gestures like clapping or putting hands together in greetings (Bagwasi, 2012; Mathoho, 2009; Nenungwi, 2015). Importantly, these gestures may overlap and be used in combination, thus serving multifunctional roles in communication (Mphasha et al., 2021).

A particularly notable cultural practice is the removal of headgear during greetings and interactions, which signifies deference to elders. This custom is not unique to the Pedi culture; it is observed in other African communities such as the Tswana, where men are expected to remove their hats in the presence of elders (Moloko-Phiri et al., 2022). According to Bagwasi (2012) and Wojtowicz (2021), as a sign of respect, Black African men observe cultural etiquette by removing their hats when interacting with senior men. Similarly, the practice of pointing at elders is considered disrespectful in many African cultures, including the Pedi culture, and could pose challenges in therapy settings where pointing may be expected (Aleksandrovna, 2021; Al-Qaderi et al., 2017). For instance, in speech-language pathology settings, Pedi children may refuse to point at objects that are close to SLPs, even if instructed to do so, because that may signify pointing at an elder, and thus disrespectful according to their culture.

### The role of greetings in the Pedi culture

Greetings are a vital aspect of social interactions in the Pedi culture. Respect in greetings is conveyed through both verbal expressions and physical actions. Younger people often use gestures such as clapping, kneeling, removing headwear or bowing, paired with verbal expressions to show deference to their elders (Bagwasi, 2012; Moloko-Phiri et al., 2022). In the Pedi culture, greetings are formal and use the plural form to refer to individuals, underscoring the respect and reverence shown to elders. This practice aligns with other African cultures, where younger generations are expected to greet elders using plural terms, even when referring to an individual elder (Mphasha et al., 2021; Ndhlovu, 2018).

In the Pedi society, greetings are primarily initiated by younger individuals. However, there are nuances depending on the specific community. For instance, in the Bangwaketse Tswana tribe, younger generations must greet their elders when they come across them on the streets, but if the older person visits their home, they (i.e. elder) should initiate the greetings (Bagwasi, 2012). In the Bangwato Tswana tribe, greetings are always initiated by the younger generations, even when the older person is a guest in their homes (Bagwasi, 2012). In line with the sentiments expressed by Moloko-Phiri et al. (2022), African clients from cultures where it is customary for younger generations to greet their elders may anticipate that SLPs who are younger than them will do the same. In contrast, some of the participants of this research relayed that initiation of greetings is not solely the responsibility of the younger generations. Akindele (2007) found that elders in the Sotho culture initiated greetings more than the younger people. Bagwasi (2012) noted that these days, younger people within the Bangwaketse Tswana tribe expect elders to greet them, not vice versa. Wojtowicz (2021) also noted that compared to rural areas, younger people who move to urban areas are less likely to greet their elders. Pedi elders are usually the ones who ask younger people about their well-being during a greeting exchange. Contrary to this, Bagwasi (2012) revealed that younger people of the Bangwaketse Tswana tribe do not mind asking their elders about their well-being. This is however something new to the Bangwaketse Tswana tribe, which Bagwasi (2012) believes was brought about by the interactions that the younger generations have had with people from other cultures. The dynamic nature of these cultural practices is important for SLPs to consider, especially in cross-cultural interactions.

### Addressing elders and kinship terms

In the Pedi culture, it is customary for younger people to avoid using the names of elders. Instead, they use kinship terms like ‘father’, ‘mother’, ‘uncle’ or ‘aunt’ as a mark of respect. This practice aligns with other African cultures such as the Zulu culture where addressing elders by their names is discouraged (Ntuli, 2012). These kinship terms are an essential part of everyday communication and are not only limited to family members but extend to teachers, elders and other figures of authority (Mulyanah & Krisnawati, 2022). The use of plural forms when addressing elders further reinforces the respectful tone of communication in the Pedi culture. For example, even if a child is referring to a single elder, they may address them in the plural form, signifying respect, even though it is grammatically incorrect (Bagwasi, 2012; Hellen, 2018). This custom can be crucial in a speech-language pathology context. Pedi children may address SLPs using familial terms or may exhibit the plural form of speech when referring to a single adult. This practice may be reflected in activities like picture naming, where a child might refer to an image of a man or woman as ‘fathers’ or ‘mothers’ instead of singular terms. Understanding and accepting these linguistic nuances may be essential for creating effective communication and rapport with Pedi clients. SLPs can acknowledge the respectful intent behind such language use, utilise these instances as teachable moments to explore the contrast between home and school language use, particularly for older children, in a way that builds on their existing cultural knowledge. Home activities can include validation of these forms (e.g. ‘Let us talk about what you call the people in your family at home’.), which can be both linguistically enriching and culturally affirming.

### Eye contact and silence in the Pedi culture

Eye contact is another significant aspect of communication in the Pedi culture. In contrast to Western societies, where direct eye contact is a sign of engagement and respect (Aleksandrovna, 2021; Al-Qaderi et al., 2017), maintaining direct eye contact with an elder in the Pedi culture is considered disrespectful (Mncwango, 2009; Vorster et al., 2022). This practice extends to other African cultures such as the Venḓa culture, where indirect eye contact is seen as a way to show respect (Moloko-Phiri et al., 2022; Nenungwi, 2015). SLPs working with Pedi clients need to be mindful of this cultural norm. Pedi children may avoid direct eye contact during speech-language pathology sessions, which should not be misinterpreted as a lack of engagement or interest. Instead, it reflects their cultural value of respecting authority figures, particularly elders. SLPs should adapt their approach to accommodate these cultural differences, understanding that indirect eye contact may signal respect rather than a communicative challenge. SLPs can avoid emphasizing direct eye contact as a communication goal or expectation. They can also model and reinforce respectful communication by acknowledging that looking away is acceptable and expected in their culture, helping the child feel more at ease.

Silence, especially during family meals or conversations, is another way in which respect is expressed in the Pedi culture. Mmangaka-Bagwasi (2012) found that, in the Tswana culture, children are not allowed to speak during family mealtimes. Although family mealtimes have communication benefits for children (Balton et al., 2019; Jarrett et al., 2016), home programme activities around mealtimes may not be effective in Pedi families. Home programmes can encourage communication activities that fit within the child’s daily routines (e.g. naming objects while doing chores) and respect the silence observed during family mealtimes. Taking turns with elders is not allowed in Pedi families. According to Mmangaka-Bagwasi (2012), in the Tswana culture, children are expected to remain silent when elders are speaking, especially their father, a practice that signifies their lower status within the family hierarchy. Within African cultures, it is considered respectful for younger individuals to remain quiet and allow elders to speak without interruption (Mmangaka-Bagwasi, 2012; Moloko-Phiri et al., 2022; Wojtowicz, 2021). This expectation can influence communication during therapy sessions, as Pedi children may be hesitant to speak, even if prompted. SLPs need to be aware that silence does not necessarily indicate a lack of cooperation but rather a cultural expression of respect. Furthermore, in certain situations, Pedi males may expect females and younger SLPs to maintain silence when they speak, mirroring the hierarchical communication structures within their families.

### Verbal communication and initiation of conversations

In the Pedi culture, verbal communication is also governed by hierarchical and gender-specific norms. Children are not expected to initiate conversations with elders but are rather taught to wait until invited to speak. This practice could influence the way Pedi children engage in speech-language pathology sessions, as they may not take the initiative in conversations, especially with SLPs who are older. SLPs might be younger in age than the children they are working with, however, because SLPs hold a professional role, children may still perceive them as authoritative or superior, regardless of age (Paul & Norbury, 2012). Similarly, if a child attends a therapy session with a parent (where the parent is the client), they may choose to remain silent, observing the interaction without contributing, as a way of not disturbing the elders. In such cases, including those involving turn-taking, SLPs can use structured activities that provide the child with clear, respectful cues to respond (e.g., “Now it is your turn to tell me…’”), framing participation as a form of permission rather than expectation. Predictable routines can also be created where initiation is expected and practised in culturally appropriate contexts, such as greeting rituals or storytelling sequences where the child is introduced as the next speaker.

The hierarchical nature of communication in Pedi families also extends to the channels through which information is relayed. Children typically communicate with their mothers, who then pass the information on to their fathers. Gender differences further shape these communication patterns, with male children often preferring to speak with their fathers and female children with their mothers (Renzaho et al., 2011; Vilanculos & Nduna, 2017). For SLPs, this suggests the importance of understanding family dynamics and communication channels, as it may impact how information about the child’s progress is shared and who is the most effective family member to engage during therapy. In home programmes, SLPs can adapt activities to fit familial communication patterns, for instance, encourage the mother (if she is the primary communication partner) to carry out practice conversations or language use games and guide her on how to gradually increase the child’s verbal participation.

## Conclusion

The findings of this study have important implications for SLPs working with Pedi clients, particularly in terms of family-centred intervention. Family-centred intervention emphasises the role of the family in the therapeutic process, making it essential for SLPs to respect cultural norms and practices when designing intervention strategies (Paul & Norbury, 2012; Paul et al., 2018). Understanding the Pedi culture’s emphasis on respect through non-verbal gestures, hierarchical communication and avoidance of eye contact can help SLPs create more effective and culturally appropriate interventions. Additionally, SLPs must be aware of the impact of cultural norms on communication behaviours. For example, Pedi children may be reluctant to take the lead in therapy sessions (child-centred intervention), given their upbringing in a culture where conversational guidance typically comes from elders. In such cases, SLPs should adapt their approach, perhaps taking a more active role in guiding the interaction while still respecting the child’s cultural norms.

One of the key strengths of this study is its emic approach (Verbuyst, 2024). This cultural insider perspective allowed for a deeper understanding and effective communication with participants. The use of semi-structured interviews and participant observations helped gather rich, detailed data, contributing to the study’s credibility. However, the study also had limitations, such as the decision not to use video recordings during observations and the gender imbalance among participants, which could have influenced the findings.

Understanding the cultural nuances of communication in the Pedi culture, including respect through verbal and non-verbal cues, is critical for SLPs. By recognising these cultural practices as differences rather than deficits, SLPs can provide more effective, respectful and culturally appropriate interventions. The findings of this study contribute to a broader understanding of how language is used in the Pedi culture and offer valuable insights for practitioners working with clients from diverse cultural backgrounds. Moreover, these findings call for a more inclusive approach to pragmatics in speech-language pathology that acknowledges the rich diversity of communication practices worldwide.
